# Dezocine Exerts Analgesic Effects in Chronic Pain by Activation of κ- and μ-Opioid Receptors and Inhibition of Norepinephrine and Serotonin Reuptake

**DOI:** 10.1155/prm/5656675

**Published:** 2025-04-04

**Authors:** Zihan Liu, Anan Liu, Jing Chen, Jing-Rui Chai, Panwen Liu, Ru-Feng Ye, Jing-Gen Liu, Yu-Jun Wang

**Affiliations:** ^1^School of Chinese Materia Medica, Nanjing University of Chinese Medicine, Nanjing, Jiangsu 210023, China; ^2^Shanghai Institute of Materia Medica, Chinese Academy of Sciences, Shanghai 201203, China; ^3^Shandong Laboratory of Yantai Drug Discovery, Bohai Rim Advanced Research Institute for Drug Discovery, Yantai, Shandong 264117, China; ^4^School of Pharmaceutical Sciences, Zhejiang Chinese Medical University, Hangzhou, Zhejiang 310053, China

**Keywords:** analgesic, chronic pain, dezocine, norepinephrine reuptake, opioid receptor, serotonin reuptake

## Abstract

**Background:** Dezocine is a leading analgesic in China used for relieving moderate to severe pain. Previous studies have characterized its pharmacological properties, demonstrating its role as a partial agonist at both the κ-opioid receptor (KOR) and the μ-opioid receptor (MOR), thereby producing potent antinociceptive effects in acute pain models. However, its efficacy and mechanisms in chronic pain management remained unclear.

**Methods:** Chronic pain models, including chronic neuropathic pain and cancer pain, were employed using chronic constriction injury (CCI) of the sciatic nerve and bone cancer pain (BCP) methodologies, respectively. The assessment of the mechanical allodynia was conducted using a von Frey filament.

**Results:** Dezocine, administered via the intraperitoneal route, alleviated both neuropathic pain and cancer pain in a dose-dependent manner, with ED_50_ of 1.3 mg/kg and 1.6 mg/kg, respectively. In the CCI model, the analgesic effect of dezocine was significantly inhibited by pretreating with KOR antagonist nor-BNI, MOR antagonist β-FNA, α2-adrenoceptor antagonist yohimbine, and 5-HT2A receptor antagonist altanserin. In the BCP model, dezocine-induced analgesia was markedly suppressed by nor-BNI, β-FNA, and yohimbine but not altanserin.

**Conclusion:** These results suggest that, in neuropathic pain, the analgesic effects of dezocine are mediated through KOR and MOR activation, together with norepinephrine reuptake inhibition (NRI) and serotonin reuptake inhibition. In contrast, in cancer pain, KOR and MOR activation and NRI are involved in mediating the analgesic effect of dezocine. This study, along with previous data, enhances our understanding of the potential clinical utility of dezocine and elucidates its mechanisms of action in chronic pain management.

## 1. Introduction

Dezocine, a bridge aminotetralin compound, acts as a partial agonist at the μ-opioid receptor (MOR) and κ-opioid receptor (KOR) and also inhibits the norepinephrine transporter (NET) and serotonin transporter (SERT) [1–3]. It is extensively utilized in China for general anesthesia induction, postoperative analgesia, as well as preemptive and postoperative analgesia [4–8]. According to a market investigation report, dezocine holds up to 60% of the market share in narcotic analgesics within China, with revenues amounting to approximately CNY 2.06 billion in 2020. For moderate to severe postoperative pain, dezocine is as potent or more potent than morphine in providing analgesia [2, 3, 9]. However, there is a lack of conclusive evidence supporting its efficacy in treating chronic pain.

Similar to the other mixed agonist/antagonist opioids such as pentazocine, butorphanol, and buprenorphine [10], dezocine has proven to be an effective analgesic in various preclinical behavioral models addressing thermal [6, 9], mechanical [9, 11], chemical [12], inflammatory [11–13], neuropathic [14], and cancer pain models [15]. Dezocine also boasts a safer side effect profile compared to full agonist morphine, showing reduced abuse liability [2], less respiratory depression [16, 17], and lower incidence of constipation [12]. Further evidence indicates that dezocine can alleviate morphine-induced tolerance, withdrawal symptoms, and conditioned place preference behavior, highlighting its potential role in managing opioid dependence [18]. Nonetheless, the exact mechanism underlying dezocine's action remains under study, particularly in rodent models, with conflicting outcomes. Studies involving neuropathic and cancer pain models suggest that dual MOR and norepinephrine reuptake mechanisms contribute to its analgesic activity [6, 15]. However, we previously found that in a vesical pain model, dezocine's antinociceptive effects were attributed to both KOR and MOR involvement [12]. Qiao and colleagues also identified a connection between its analgesic effects and KOR activity [19]. These studies indicate dezocine's pain-relieving effects across various pain types but highlight the need for further investigation into its distinct mechanism of action.

Chronic pain poses a significant clinical challenge worldwide. Neuropathic pain, often resulting from somatosensory nervous system disorders, is difficult to treat and shows limited responsiveness to opioids [20]. Selective serotonin–norepinephrine reuptake inhibitors (NRIs) are frequently recommended as the first line of therapy for this condition [21]. Meanwhile, cancer-related bone pain, primarily caused by bone metastasis, is a common and debilitating symptom [22], for which strong opioids such as morphine are typically used, albeit with careful monitoring due to potential adverse side effects [23]. There is an urgent need for new therapeutic strategies to improve treatment outcomes and enhance patients' quality of life. Given its multiple targets, including opioid receptors, and NET–SERT, dezocine might offer potential benefits in managing chronic pain. The current study aims to (1) evaluate the analgesic effectiveness of dezocine on neuropathic and cancer pain–induced mechanical allodynia using chronic pain models such as chronic constriction injury (CCI) of sciatic nerve and bone cancer pain (BCP); and (2) explore its underlying mechanisms of action.

## 2. Methods

### 2.1. Animals

Male Sprague Dawley rats (200–220 g) and female Wistar rats (60–80 g) were obtained from Shanghai SLAC Laboratory Animal Co. Ltd. The rats were housed in a room at a controlled temperature (21°C–24ºC) and a controlled 12 h light-dark cycle (light on 07:00 a.m.) with free access to food and water. All animal experiments and procedures were approved by the Animal Care and Use Committee of Shanghai Institute of Materia Medica, Chinese Academy of Sciences (Approval no. 2022-06-LJG-68; 2023-02-LJG-22; 2023-10-WYJ-06). For all animal procedures, we adhered strictly to ethical guidelines and institutional protocols. Euthanasia was performed under anesthesia, ensuring minimal distress and discomfort.

### 2.2. Drugs

Dezocine injection solution was kindly provided by Yangzi River Pharmaceutical Group. Gabapentin was purchased from Sinopharm Chemical Reagent Co., Ltd. β-Funaltrexamine hydrochloride (β-FNA), NAD 299 hydrochloride, altanserin hydrochloride, and RS 102221 hydrochloride were purchased from TOCRIS. Norbinaltorphimine (nor-BNI) was obtained from Abcam Co., Ltd. Yohimbine was purchased from Selleckchem.

### 2.3. CCI of Sciatic Nerve–Induced Pain

The CCI of the sciatic nerve model was established using a previously described method [24, 25]. In brief, Sprague Dawley rats were anesthetized with an intraperitoneal injection of 50 mg/kg of pentobarbital sodium. Following disinfection of the skin surface, an incision was made along the midsection of the left thigh to expose the main sciatic nerve. Four 4-0 protein sutures were then tied around the nerve at 1 mm intervals. In the sham group, the sciatic nerve was similarly exposed but left unligated. After the procedure, the incision was closed with sutures. The entire surgical process was completed within 15 min.

### 2.4. Bone Cancer–Induced Pain

The BCP model was established by injecting walker 256 mammary gland carcinoma cells into the tibia, as reported previously [26–28]. Before the surgery, the walker 256 mammary gland carcinoma cells were taken out from liquid nitrogen and gently thawed in a 37°C water bath. Subsequently, 0.5 mL of these dissolved ascites cells were injected into the abdominal cavity of 2 female Wistar rats (70 g). After a period of 7 days, the ascites fluid was harvested and centrifuged at 400 × g for 10 min. The supernatant was collected and washed 2–4 times and adjusted to an appropriate concentration of 1 × 10^8^/mL, and then stored on ice for further use.

For the surgical procedure, the rats were first anesthetized with an intraperitoneal injection of 50 mg/kg of pentobarbital sodium, and the right tibial tubercle was exposed. A 23-gauge needle was used to create an opening in the bone marrow cavity, into which 5 μL of the walker 256 cell suspension was slowly injected. The wound was disinfected after the injection.

### 2.5. Behavioral Assessment of Mechanical Allodynia

The assessment of the mechanical allodynia was conducted using a von Frey filament [29, 30]. The rats were placed in a transparent metal grid cager (22 cm length, 22 cm width, and 13.3 cm height) and allowed to acclimate for 15 min until no probing behaviors were observed. A series of increasing forces (0.6, 1.0, 1.4, 2, 4, 6, 8, 10, and 15 g) was applied to stimulate the right hind limb, eliciting a withdrawal response. A force of 2 g was first used to stimulate the left hind toe, administered for five consecutive times, each lasting for 10 s, with 1 min intervals between stimulations. If the paw withdrawal response occurred fewer than three times, the force was increased to 4 g. Conversely, if the withdrawal occurred three or more times, the subsequent force of 1.4 g was chosen. The minimal force that resulted in more than three paw withdrawal reactions was defined as the mechanical stimulus paw withdrawal threshold (PWT). If the necessary force exceeded 15 g or was below 0.6 g, it was directly recorded as 15 or 0.6 g, respectively.

### 2.6. Statistical Analysis

All data were presented as means ± SEM. Data analysis was performed using GraphPad Prism 7.0 by two-way ANOVA with Bonferroni's post hoc test. Values of ⁣^∗^*p* < 0.05 were considered statistically significant.

## 3. Results

### 3.1. Establishment of Neuropathic Pain and Cancer Pain Models

**Figure 1:**
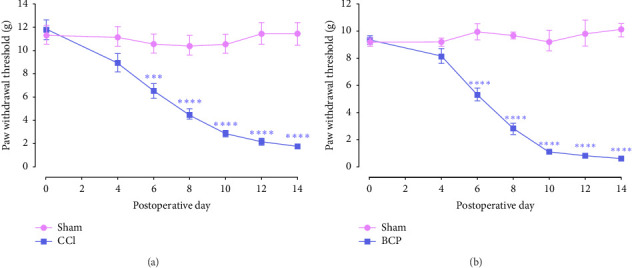
Time course of mechanical allodynia after CCI and BCP surgeries in rats. Changes of mechanical threshold after CCI (a) and BCP (b). Data were presented as the mean ± SEM (*n* = 12). ⁣^∗∗∗^*p* < 0.001 and ⁣^∗∗∗∗^*p* < 0.0001 compared to the sham group, by two-way ANOVA followed by Bonferroni's post hoc test.

The establishment of neuropathic and cancer pain models was achieved using the chronic compression injury of the sciatic nerve and metastatic BCP models, both of which are widely used in chronic pain research. Previous studies demonstrated that following either chronic compression injury of the sciatic nerve surgery or transplantation of walker 256 mammary gland carcinoma cells into the tibia, rats develop mechanical allodynia, which can be quantitatively measured using von Frey filaments to determine the PWT. As shown in Figure 1(a), on day 6 post-CCI surgery, the PWT in the CCI group was significantly lower compared to the sham group, and this reduction persisted until Day 14 (*F* [6, 154 = 13.57, *p* < 0.0001; two-way ANOVA with Bonferroni's post hoc test). In Figure 1(b), rats with BCP also displayed mechanical hypersensitivity with significantly decreased PWT in comparison to the sham group (*F* [6, 154] = 34.3, *p* < 0.0001, two-way ANOVA followed by Bonferroni's post hoc test). The PWT stabilized by Day 10 postsurgery. These data indicated that both CCI and BCP models successfully induce mechanical allodynia in rats.

### 3.2. Dezocine Produced Significant Analgesic Effects in Both Chronic Pain Models

**Figure 2:**
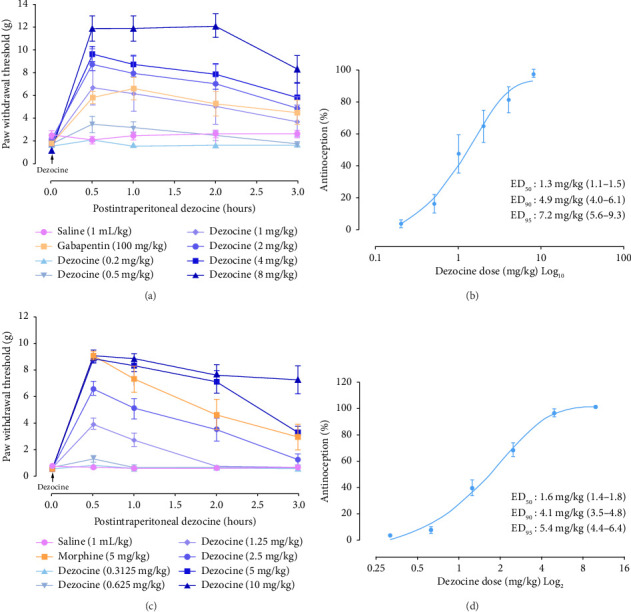
Dezocine produced dose-dependent analgesic effects in CCI and BCP models. Time courses (a and c) and dose-dependent curves (b and d) for the effects of dezocine. After intraperitoneal injection of various doses of dezocine and reference gabapentin or reference morphine, respectively, changes in paw withdrawal threshold (PWT) were monitored. The analgesic ED_50_ value was calculated from the data obtained at 0.5 h after injection. Data were presented as the mean ± SEM (*n* = 8–14).

Utilizing these models, we investigated the analgesic effects of dezocine. Animals were injected with dezocine by a systemic intraperitoneal route at various doses. As presented in Figure 2(a), dezocine increased the PWT induced by CCI in a dose-dependent manner, reaching peak effects 0.5 h after injection. The ED_50_ for dezocine in relieving CCI–induced neuropathic pain was 1.3 mg/kg (Figure 2(b)). Similarly, dezocine increased the PWT in the BCP model dose-dependently, with peak effects occurring 0.5 h after administration (Figure 2(c)). The ED_50_ for its analgesic effect in the cancer pain model was 1.6 mg/kg (Figure 2(d)). Overall, these data indicate that dezocine produces significant analgesic effects against mechanical allodynia induced by CCI and BCP.

### 3.3. Both MOR and KOR Antagonists Inhibited the Analgesic Effects of Dezocine

**Figure 3:**
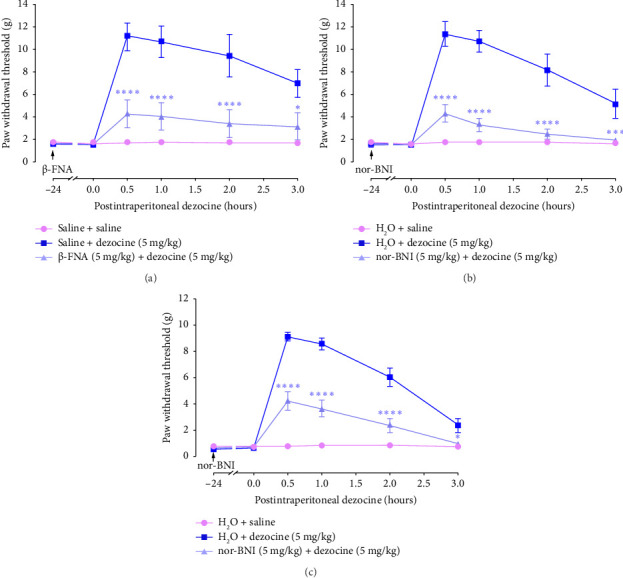
KOR and MOR antagonists significantly reduced the analgesic effects of dezocine. (a) Effects of MOR antagonist β-FNA on dezocine-induced analgesic effects in CCI. (b and c) Effects of KOR antagonist nor-BNI on dezocine-induced analgesic effects in CCI (b) and BCP (c). Rats were subcutaneously pretreated with 5 mg/kg β-FNA or 5 mg/kg nor-BNI for 24 h, and then intraperitoneally injected with 5 mg/kg dezocine. Changes in PWT after dezocine injection were monitored. Data were presented as the mean ± SEM (*n* = 8–11). ⁣^∗^*p* < 0.05, ⁣^∗∗∗^*p* < 0.001, and ⁣^∗∗∗∗^*p* < 0.0001 compared to the dezocine group, by two-way ANOVA followed by Bonferroni's post hoc test.

To identify which opioid receptor subtypes are involved in the analgesic effects of dezocine on neuropathic and BCP, we first used MOR antagonist β-FNA. β-FNA was administered subcutaneously at a dose of 5 mg/kg [31], and 24 h later, the analgesic effects of dezocine were evaluated. It was found that β-FNA significantly inhibited the increase in PWT induced by dezocine in the CCI model (*F* [10, 144] = 5.093, *p* < 0.0001, two-way ANOVA with Bonferroni's post hoc test; Figure 3(a)). Subsequently, we assessed the effect of KOR antagonist nor-BNI on dezocine-induced action. nor-BNI was administered subcutaneously at 5 mg/kg [32], and its effects were assessed 24 h later. As shown in Figures 3(b) and 3(c), nor-BNI markedly attenuated dezocine-induced increase in PWT in both the CCI model (*F* [10, 144] = 12.8, *p* < 0.0001, two-way ANOVA followed by Bonferroni's post hoc test; Figure 3(b)) and BCP model (*F* [10,180] = 27.97, *p* < 0.0001, two-way ANOVA with Bonferroni's post hoc test; Figure 3(c)). These data indicate that the analgesic effects of dezocine on chronic pain are mediated through the activation of both the KOR and MOR.

### 3.4. α2-Adrenoceptor Antagonist Yohimbine Suppressed Dezocine-Induced Analgesic Effects

**Figure 4:**
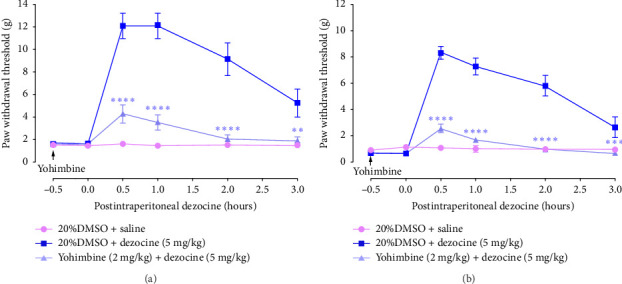
*α*2-adrenoceptor antagonist yohimbine significantly suppressed the analgesic effects of dezocine. (a and b) Effects of yohimbine on dezocine-induced analgesic effects in CCI (a) and BCP (b). Rats were intraperitoneally pretreated with 2 mg/kg yohimbine for 0.5 h, and then intraperitoneally injected with 5 mg/kg dezocine. Changes in PWT after dezocine injection were monitored. Data were presented as the mean ± SEM (*n* = 8). ⁣^∗∗^*p* < 0.01, ⁣^∗∗∗^*p* < 0.001, and ⁣^∗∗∗∗^*p* < 0.0001 compared to the dezocine group, by two-way ANOVA followed by Bonferroni's post hoc test.

Given that the NET is a newly identified molecular target of dezocine, we determined the effect of *α*2-adrenoceptor antagonist yohimbine on dezocine-induced analgesia. Yohimbine was administered intraperitoneally at a dose of 2 mg/kg [33], and 0.5 h later, the analgesic effects of dezocine were assessed. As shown in Figures 4(a) and 4(b), yohimbine significantly attenuated dezocine-induced increase in PWT in both the CCI model (*F* [10, 126] = 15.39, *p* < 0.0001, two-way ANOVA with Bonferroni's post hoc test; Figure 4(a)) and BCP model (*F* [10, 126]  = 25.91, *p* < 0.0001, two-way ANOVA with Bonferroni's post hoc test; Figure 4(b)). The results suggest that dezocine-induced analgesic effects in neuropathic and cancer pain models are also mediated by the inhibition of norepinephrine reuptake.

### 3.5. 5-HT2A Receptor Antagonist Altanserin Attenuated Dezocine-Induced Analgesic Effects in Neuropathic Pain but Not in Cancer Pain

**Figure 5:**
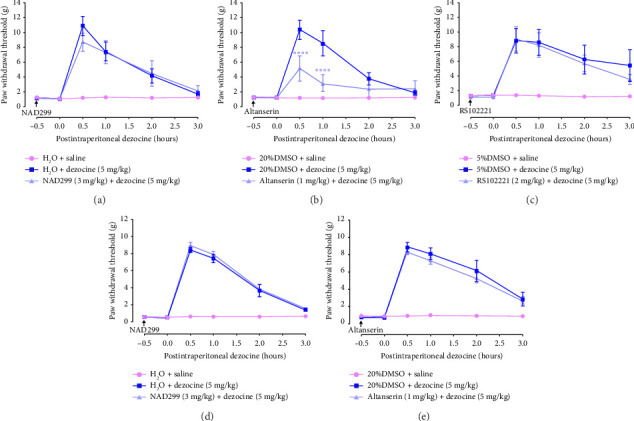
5-HT2A antagonist altanserin markedly inhibited the analgesic effects of dezocine in CCI but not BCP. (a–c) Effects of 5-HT1A antagonist NAD299 (a), 5-HT2A antagonist altanserin, (b) and 5-HT2C antagonist RS102221 (c) on dezocine-induced analgesic effects in CCI. (d and e) Effects of 5-HT1A antagonist NAD299 (d) and 5-HT2A antagonist altanserin (e) on dezocine-induced analgesic effects in BCP. Rats were subcutaneously pretreated with 3 mg/kg NAD299, 1 mg/kg altanserin or intraperitoneally pretreated with 2 mg/kg RS102221 for 0.5 h, then intraperitoneally injected with 5 mg/kg dezocine. Changes in PWT after dezocine injection were monitored. Data are presented as the mean ± SEM (*n* = 8). ⁣^∗∗∗∗^*p* < 0.0001 compared to the dezocine group, by two-way ANOVA followed by Bonferroni's post hoc test.

Beyond its interaction with the norepinephrine transporter, dezocine also targets the serotonin transporter. To explore the role of serotonin receptors, we examined the effects of various antagonists: 5-HT1A receptor antagonist NAD299 (administered subcutaneously at 3 mg/kg) [34], 5-HT2A receptor antagonist altanserin (administered subcutaneously at 1 mg/kg) [35], and 5-HT2C receptor antagonist RS102221 (administered intraperitoneally at 2 mg/kg) [36]. Dezocine-induced analgesic effects were evaluated 0.5 h postadministration. As shown in Figures 5(a), 5(b), and 5(c), altanserin significantly attenuated dezocine-induced increase in PWT in the CCI model (*F* [10, 126] = 6.328, *p* < 0.0001, two-way ANOVA with Bonferroni's post hoc test; Figure 5(b)), however, NAD299 and RS102221 did not exhibit this effect. In the BCP model, neither altanserin nor NAD299 affected the analgesic effects of dezocine (Figures 5(d) and 5(e)). These results suggest that the serotonin reuptake mechanism plays a role in dezocine-induced analgesic effects on neuropathic pain, but this mechanism does not appear to contribute to its analgesic effects in cancer pain.

## 4. Discussion and Conclusion

In the present study, we demonstrated that dezocine exhibited significant analgesic effects on mechanical allodynia induced by both CCI and BCP. These effects are mediated through the activation of KORs and MORs, in addition to NRI and serotonin reuptake inhibition. Notably, in chronic cancer pain, the analgesic effect of dezocine involves KOR and MOR activation and NRI, while serotonin reuptake inhibition is not a contributing factor. A novel aspect of this research is the differential mechanisms employed by dezocine in neuropathic vs. cancer pain. Another significant finding is the recognition of dezocine's partial agonist activity at the KORs, which contrasts with prior studies that primarily noted its KOR antagonist properties.

Dezocine is well identified as a partial agonist of the MOR and used for pain management [1, 3]. Our previous research indicated that the antinociceptive effects of dezocine in the abdominal constriction test were a result of action at MOR [12]. This study corroborates the finding, demonstrating that the MOR antagonist β-FNA significantly attenuated dezocine-induced analgesic effects in both CCI and BCP–induced mechanical allodynia. This was in accordance with recent findings that dezocine's analgesic effects in neuropathic and cancer pain models are partially mediated through MOR activation [6, 15].

Our findings also indicate that dezocine's analgesic effects in the CCI and BCP models are due to KOR activation, as indicated by the significant inhibition of dezocine's effects by the KOR antagonist nor-BNI. These results were consistent with our earlier findings that nor-BNI markedly suppressed dezocine-induced antinociception in the acute abdominal constriction test [12]. nor-BNI is a widely recognized selective KOR antagonist [37], and other studies have also shown that it can inhibit the antinociceptive effects of dezocine in the hot plate and abdominal constriction tests [19]. However, there is some controversy, as some studies indicate that dezocine might act as a KOR antagonist, unable to activate G protein and produce functional effects [1, 38]. Wang et al. [6] found no modulation of mechanical antiallodynia induced by dezocine when pretreated with either KOR antagonist GNTI or nor-BNI. These differences may be attributed to the diverse models of neuropathic pain, routes of administration, and forms of nor-BNI used. Wang with colleagues used a spared nerve injury (SNI) of neuropathic pain to examine the antihypersensitivity activity of dezocine, whereas, in this study, we adapted the CCI model of neuropathic pain. Nor-BNI was administrated with the subcutaneous route in this study; however, GNTI and nor-BNI were given intrathecally in a study from Wang et al. [6].

Following the discovery by Liu et al. [1] that the NET and SERT are novel molecular targets of dezocine, the interest in its applications and mechanisms has increased. Our findings align with previous reports that yohimbine, a selective *α*2-adrenoceptor antagonist, significantly reduces dezocine-induced analgesic effects in both neuropathic and BCP[6]. More importantly, we found that subcutaneous injection of 5-HT2A antagonist altanserin, but not 5-HT1A antagonist NAD299 or 5-HT2C antagonist RS102221, significantly inhibited dezocine-induced analgesic effects in the CCI model. Serotonin reuptake inhibition may cause increased serotonin in the brain, activate 5-HT receptors, and consequently contribute to dezocine-induced analgesic effects. Indeed, 5-HT and its receptors are widely known to be involved in pain modulation [39, 40]. However, in the BCP model, 5-HT2A antagonist altanserin did not affect dezocine's analgesic effects. Thus, these findings indicate that while norepinephrine reuptake contributes to dezocine's analgesic effects in both types of pain, serotonin reuptake plays a role only in neuropathic pain. This difference may be due to norepinephrine's critical role in pain regulation, with norepinephrine reuptake inhibitors being particularly effective in managing neuropathic and chronic pain [41–44].

In conclusion, dezocine exhibits marked analgesic effects on mechanical allodynia induced by neuropathic and cancer pain. It achieves these effects through actions on KOR, MOR, NET, and SERT, with mechanisms varying slightly depending on the type of pain. These findings suggest that dezocine could be a promising candidate for chronic pain treatment.

## Data Availability

All data needed to evaluate the conclusions in the paper are presented in the results. Any additional information is available from the corresponding author.
